# Protein-altering and regulatory genetic variants near *GATA4* implicated in bicuspid aortic valve

**DOI:** 10.1038/ncomms15481

**Published:** 2017-05-25

**Authors:** Bo Yang, Wei Zhou, Jiao Jiao, Jonas B. Nielsen, Michael R. Mathis, Mahyar Heydarpour, Guillaume Lettre, Lasse Folkersen, Siddharth Prakash, Claudia Schurmann, Lars Fritsche, Gregory A. Farnum, Maoxuan Lin, Mohammad Othman, Whitney Hornsby, Anisa Driscoll, Alexandra Levasseur, Marc Thomas, Linda Farhat, Marie-Pierre Dubé, Eric M. Isselbacher, Anders Franco-Cereceda, Dong-chuan Guo, Erwin P. Bottinger, G. Michael Deeb, Anna Booher, Sachin Kheterpal, Y. Eugene Chen, Hyun Min Kang, Jacob Kitzman, Heather J. Cordell, Bernard D. Keavney, Judith A. Goodship, Santhi K. Ganesh, Gonçalo Abecasis, Kim A. Eagle, Alan P. Boyle, Ruth J. F. Loos, Per Eriksson, Jean-Claude Tardif, Chad M. Brummett, Dianna M. Milewicz, Simon C. Body, Cristen J. Willer

**Affiliations:** 1 Department of Cardiac Surgery, University of Michigan, Ann Arbor, Michigan 48109, USA; 2 Frankel Cardiovascular Center, University of Michigan, Ann Arbor, Michigan 48109, USA; 3 Department of Computational Medicine and Bioinformatics, University of Michigan, Ann Arbor, Michigan 48109, USA; 4 Division of Cardiovascular Medicine, Department of Internal Medicine, University of Michigan, Ann Arbor, Michigan 48109, USA; 5 Department of Anesthesiology, University of Michigan, Ann Arbor, Michigan 48109, USA; 6 Department of Anesthesiology, Perioperative, and Pain Medicine, Brigham and Women's Hospital, Harvard Medical School, Boston, Massachusetts 02115, USA; 7 Montreal Heart Institute, Montreal, Quebec, Canada HIT 1C8; 8 Department of Medicine, Université de Montréal, Montreal, Quebec, Canada QC H3T 1J4; 9 Cardiovascular Medicine Unit, Center for Molecular Medicine, Department of Medicine, Karolinska University Hospital Solna, Karolinska Institutet, Stockholm SE-171 76, Sweden; 10 Center for Biological Sequence Analysis, Technical University of Denmark, Copenhagen DK-2800, Denmark; 11 Department of Internal Medicine, Division of Medical Genetics, University of Texas Health Science Center at Houston McGovern Medical School, Houston, Texas 77030, USA; 12 The Charles Bronfman Institute for Personalized Medicine, The Icahn School of Medicine at Mount Sinai, New York, New York 10029, USA; 13 Department of Biostatistics, University of Michigan, Ann Arbor, Michigan 48109, USA; 14 Norwegian University of Science and Technology, Trondheim 7491, Norway; 15 Department of Ophthalmology and Visual Sciences, University of Michigan, Ann Arbor, Michigan 48105, USA; 16 Cardiothoracic Surgery Unit, Department of Molecular Medicine and Surgery, Karolinska University Hospital Solna, Karolinska Institutet, Stockholm SE-171 76, Sweden; 17 Department of Human Genetics, University of Michigan, Ann Arbor, Michigan 48109, USA; 18 Institute of Genetic Medicine, Newcastle University, Newcastle Upon Tyne NE1 3BZ, UK; 19 Division of Cardiovascular Sciences, Faculty of Biology, Medicine and Health, The University of Manchester, Manchester M13 9PL, UK; 20 Manchester Heart Centre, Central Manchester University Hospitals NHS Foundation Trust, Manchester M13 9WL, UK; 21 The Mindich Child Health Development Institute, The Icahn School of Medicine at Mount Sinai, New York, New York 10029, USA

## Abstract

Bicuspid aortic valve (BAV) is a heritable congenital heart defect and an important risk factor for valvulopathy and aortopathy. Here we report a genome-wide association scan of 466 BAV cases and 4,660 age, sex and ethnicity-matched controls with replication in up to 1,326 cases and 8,103 controls. We identify association with a noncoding variant 151 kb from the gene encoding the cardiac-specific transcription factor, GATA4, and near-significance for p.Ser377Gly in *GATA4*. *GATA4* was interrupted by CRISPR-Cas9 in induced pluripotent stem cells from healthy donors. The disruption of *GATA4* significantly impaired the transition from endothelial cells into mesenchymal cells, a critical step in heart valve development.

## Abstract (web summary)

Bicuspid aortic valve (BAV) is the most common human congenital cardiovascular malformation. Here, the authors perform a genome-wide association study for BAV and identify risk variants in the gene region of cardiac-specific transcription factor GATA4 and implicate GATA4 in heart valve development.

Bicuspid aortic valve (BAV) is a congenital aortic valve defect characterized by fusion of two of the normal three leaflets. With a prevalence of ∼1% in the population and a feature of some rare connective-tissue syndromes, BAV is the most common cardiovascular malformation in humans^1^,^2^. BAV is associated with serious consequences: 30–70% of those with BAV will develop dilated thoracic aorta^3^; 15–71% of BAV patients develop aortic valve stenosis depending on age group and individuals with BAV have a 50-fold higher risk of severe aortic valve stenosis^4^; and up to 47% of BAV patients develop aortic valve incompetence^5^. The presence of a BAV confers an eightfold increased risk of aortic dissection, which carries very high mortality^6^. Overall, 27% of BAV patients will require surgical intervention to either replace their aortic valve or aorta for aortic aneurysm and dissection^7^. BAV accounts for ∼40% of the >50,000 aortic valve replacements performed in the United States each year^8^.

BAV is moderately heritable, with estimates ranging from 20 to 89% (refs 9, 10, 11). Despite the prevalence, importance and heritability of BAV, its genetic origins remain elusive. Previous genetic studies of BAV have focused primarily on linkage analysis in families^9^,^12^ or sequencing candidate genes in cases^13^ under a hypothesis of Mendelian inheritance. Only one previous genome-wide association study (GWAS) for BAV has been published in a limited number of cases (*n*=68; ref. 14), which did not identify any genome-wide significant results. The only gene in which variants have been identified to cause BAV in multiple families is *NOTCH1*, but <6% of BAV cases are accounted for by *NOTCH1* variation^13^. It is clear that BAV is not a simple Mendelian trait^9^,^15^, but is indeed heritable, and, therefore, we applied genetic association methods typically used for complex traits.

With a goal of identifying genetic variants associated with BAV, leading to biological insight of the underlying causes, here we perform an unbiased genome scan in a large study of BAV cases (*n*=466) and controls (*n*=4,660), with replication in additional samples of up to 1,326 cases and 8,103 controls. We identify two genetic variants that reached or were near genome-wide significance levels (*P*<5 × 10^−8^). One is a low-frequency intergenic variant rs6601627 (odds ratio (OR)=2.38, *P*_after-replication_=3 × 10^−15^) with a substantially higher frequency in BAV cases (8.3%) than in controls (4.2%), and the other one is an independent association signal at a common protein-altering variant p.Ser377Gly (rs3729856) in *GATA4,* which encodes a cardiac-specific transcription factor that is 151 kilobases(kb) away from the first variant (*P*_after-replication_=8.8 × 10^−8^). Induced pluripotent stem cells (iPSCs) with *GATA4* disrupted by CRISPR-Cas9 demonstrate impaired transition of endothelial into mesenchymal cells (EndoMT), a critical step in valve formation^16^.

## Results

### GWAS of BAV

To discover the underlying genetic basis of BAV, we successfully genotyped 498,075 genetic variants with enrichment of protein-altering variants (43.8% of variants examined) for 466 BAV cases and 4,660 controls. Imputation from the Haplotype Reference Consortium (HRC) panel^17^ enabled examination of a total of 12,320,487 variants. Clinical characteristics for BAV cases are summarized in Supplementary Table 1. Following a genome-wide association scan (Supplementary Figs 1 and 2), we examined three variants in replication cohorts with a combined total of up to 1,326 additional cases and 8,103 controls.

### Variants near *GATA4*

**Figure 1:**
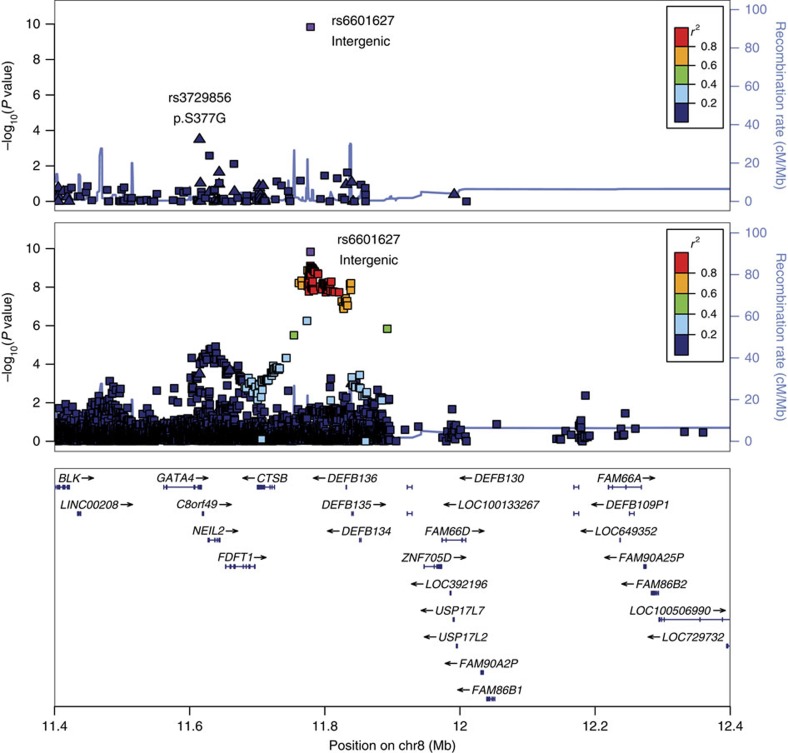
Regional association plot of the chr8 association region near *GATA4*n the discovery cohort. Genome-wide single variant association tests were performed on 466 BAV cases and 4,660 controls. The upper panel shows all variants that were directly genotyped in the chip array in this region. A missense variant (rs3729856, p.S377G) within *GATA4* was observed to be associated with BAV with *P*=3.2 × 10^−4^, that reached *P*=8.8 × 10^−8^ following replication in 1,326 BAV cases and 8,103 controls. The bottom panel shows results after genotypes imputed from the HRC reference^17^. Coding variants are represented by triangles and noncoding variants are represented by squares.

The strongest result from the genome-wide discovery for BAV was observed for a genotyped low-frequency variant, rs6601627, in an intergenic region of chromosome 8 (rs6601627, minor allele frequency (MAF)=4.1%, OR=1.9, *P*_combined_=3.0 × 10^−15^; Table 1, Fig. 1 and Supplementary Fig. 2). Ninety-seven imputed variants in this region also reached genome-wide significance (*P*<5 × 10^−8^). The two nearest genes are not obvious functional candidates (*CTSB* and *DEFB135*); however, this variant is 151 kb from the 3′ end of the *GATA4* gene (Fig. 1).

We also observed a common missense variant p.Ser377Gly in *GATA4* (rs3729856) that was also associated with BAV (*P*_discovery_=3.2 × 10^−4^, MAF=14.5%; Table 1 and Fig. 1). We selected *GATA4* p.Ser377Gly for *in silico* replication because it is a protein-altering variant and because it was located in the local genomic region of the most significant variant (Supplementary Fig. 3). The p.Ser377Gly variant reached near genome-wide significance after including *in silico* replication data (OR=1.31, *P*=8.8 × 10^−8^; Supplementary Fig. 4), and exceeded the typical significance level used for exome-wide studies of coding variation (typically *P*<2 × 10^−7^; ref. 18). This suggests that *GATA4* may be the functional gene at this GWAS locus; however, further experiments will be needed to demonstrate which gene(s) causes BAV. The two variants at 8p23.1 (rs6601627 and rs3729856) appear to be independent of each other, since they were not in linkage disequilibrium (LD *r*^2^=0.013) and reciprocal conditional association analysis maintained nominal significance for both (*P*_cond_ rs6601627=8.92 × 10^−9^, *P*_cond_ rs3729856=0.012). After including *in silico* replication data, the reciprocal conditional association analysis still maintained nominal significance (*P*_meta_ rs6601627=1.52 × 10^−9^, *P*_meta_ rs3729856=8.17 × 10^−3^; Supplementary Fig. 5). The non-additive association tests showed that both variants appear to have dominant effects on risk of BAV (Supplementary Table 2).

The ExAC database characterizes protein-altering variants in 60,706 multiethnic individuals with whole-exome sequences^19^. ExAC lists 96 missense variants in *GATA4* (95 of them have MAF<1%), a deficit compared to the 140 variants predicted based on gene size. In addition, 9.4 loss-of-function (LoF) variants are predicted and only 1 was observed (p.Lys365Ter) out of 60,706 individuals with deep exome sequences. The probability that the gene is intolerant to LoF, a measure of the relative importance of gene function, is high (loss intolerance probability (pLI) score=0.8 where >0.9 is considered extremely intolerant). Moreover, the missense *GATA4* variant rs3729856 is predicted as benign or tolerated by PolyPhen-2 (20) and SIFT^21^, and it has a combined annotation dependent depletion (CADD) score 9.418 (in top 11% of deleterious variants in the human genome)^22^. These results suggest the importance of the *GATA4* gene's function, although the missense variant rs3729856 itself may not be significantly deleterious.

We hypothesized that the functional BAV gene at this *CTSB/GATA4* locus would demonstrate high expression in heart or vascular tissue. Using the GTEx portal^23^, we examined mRNA expression levels of all genes within the 200 kb surrounding the noncoding associated variant rs6601627 and found that *GATA4* showed strong expression in the heart (atrial appendage and left ventricle) and coronary artery, and also in the ovary, testis, pancreas and liver (Supplementary Fig. 6)^23^. The other genes in the region (*NEIL2*, *FDFT1* and *CTSB*) showed ubiquitous expression levels across all tissues (Supplementary Fig. 6). Examination of all GTEx association results did not identify any significant expression quantitative trait locus with the noncoding rs6,601,627 (*P*<10^−5^; ref. 23). We propose that this noncoding variant, or a variant tagged by it, influences *GATA4* expression in a manner not detectable by GTE—either exerting an influence on gene expression levels only in the developing fetal heart or with a relatively modest effect that was not detectable in the current GTEx sample size.

After detecting the association with both coding and noncoding variants at the *GATA4* locus, we sought to examine the role of GATA4 in the development of the aortic valve. In the primitive heart tube, heart valves develop from endocardial cushions, which are formed by mesenchymal cells derived from endothelial cells (ECs) through a process called EndoMT^16^. Despite the critical role in heart valve development, the mechanism of EndoMT is not well understood^24^,^25^,^26^. GATA4 was previously shown to be essential for heart formation and for endocardial cushion development in mice^27^,^28^,^29^. Here we evaluated the impact of disruption of GATA4 on human iPSC differentiation into mesenchymal cells through EndoMT to examine the role of GATA4 in the development of aortic valves in humans.

The *GATA4* knockout mouse is embryonically lethal between embryonic day (E) 7.0 and E9.5, and lacks a primitive heart tube^28^. Deletions of *GATA4* in humans have been associated with congenital heart defects (CHDs)^30^,^31^, and a missense variant p.Gly296Ser was identified in a family with atrial and ventricular septal defects^32^. A mouse model of the p.Gly296Ser missense change is also embryonically lethal by E11.5, but a subset of these mice demonstrate semilunar valve stenosis and small defects of the atrial septum, thought to be resultant from defects in cardiomyocyte proliferation during embryogenesis^33^. Previous studies observed missense variants in *GATA4* in patients with septal defects^34^, CHDs^35^ and Tetralogy of Fallot (ToF)^35^, but have not been tested in case–control models. The frequency of *GATA4* variants in healthy controls is not clear from these studies and their pathogenicity is unknown. The co-appearance of congenital heart disease and testicular anomalies was found in a family with a *GATA4* p.Gly221Arg mutation, thought to disrupt interaction with FOG2 and/or NR5A1, important factors for gonadal development^36^. *GATA5* has 46% homology with *GATA4*. *GATA5* sequence variants have been identified in humans with BAV^37^,^38^, and *GATA5* knockout mice and zebrafish demonstrate high rates of cardiac abnormalities^39^.

### Chromatin conformation at the *GATA*4 locus

We attempted to evaluate the hypothesis that noncoding variants in LD with rs6601627 have an impact on expression of *GATA4* during a critical stage of development. This is supported by prior evidence that GATA4 dosage has an impact on cardiac formation^40^. We first identified potentially functional variants using RegulomeDB and HaploReg in the region near rs6601627 or variants in high LD (*r*^2^>0.6; ranging from rs112197605 to rs117851931; hg19:chr8:11774952–11838697)^41^,^42^. After examining local chromatin states, DNase-hypersensitive regions and transcription factor-binding sites, we identified rs118065347 as a variant likely to be functional because it co-localizes with binding regions for multiple transcription factors (including KAP1, CCNT2, CJUN, C-MYC, GATA2, HDAC2, HMGN3, JUND, MAX, SP1, TAL1, YY1 and ZBTB7A) and is in a known enhancer active in fetal heart, left and right ventricle, right atrium, as well as other tissues^42^,^43^. This variant disrupts the binding motif for a variety of transcription factors including PAX6 (ref. 44). There are other candidate functional variants in high LD with the index variant, and molecular experiments will be required to definitively identify the functional variant(s) and the mechanism of action on aortic valve development.

**Figure 2:**
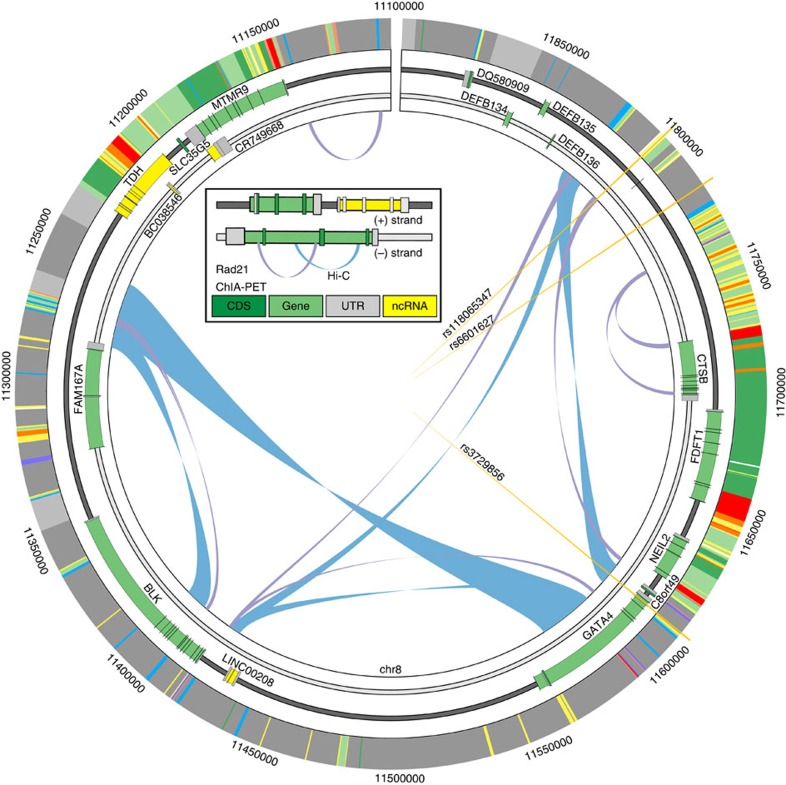
Chromatin interactions between associated variants and *GATA4*. The topological domain region containing associated variants (orange vertical lines), genes (green bars), chromatin interactions by Hi-C (blue loops) and ChIA-PET (purple loops), and chromatin state (outer ring and standard colours from ref. 68 but of significance here: yellow as enhancers, red as promoters, green as transcribed, blue as CTCF and grey as inactive). All data are from K562 cells. rs3729856 is indicated as falling within a coding exon of *GATA4*. rs6601627 was identified as the associated variant to BAV and rs118065347 is the putative functional variant in linkage. rs11865347 overlaps an annotated enhancer as well as a ChIA-PET loop connecting to a region 3′ of *GATA4*.

We next asked which genes in the locus may interact with this candidate enhancer region. We identified chromatin interaction loops in K562 and GM12878 cells using chromatin interaction analysis by paired-end tag sequencing (ChIA-PET) and high-throughput sequencing (Hi-C) data (Fig. 2)^45^,^46^. rs118065347 falls near the edge of a topologically associated domain spanning from hg19:chr8:11250000 to 11825000 defined by Hi-C in both cell lines. The variant falls inside a ChIA-PET loop connecting to a region also annotated as an enhancer 3′ of *GATA4* and C8orf49 and 5′ of *NEIL2*. These data indicate that this distal region is brought in close proximity to *GATA4* and disruption of this region may have direct impact on *GATA4* expression. Further molecular experiments will be needed to clarify the gene(s) that has an impact on BAV.

### Phenotypic characteristics of BAV cases in discovery sample

Among our 466 non-syndromic BAV cases, 93 (20%) reported one or more family members also having BAV (Supplementary Table 1). This suggests a high recurrence risk and supports the hypothesis of large-effect variants, but not necessarily Mendelian inheritance^47^. Majority of BAV cases were recruited from a cardiac surgery clinic at the University of Michigan Frankel Cardiovascular Center (FCVC), where patients are referred to cardiac surgery for aneurysm repair or valve replacement; thus, we found a high proportion of patients with thoracic aortic aneurysm (TAA; 83%). However, at these two loci, we saw no evidence for heterogeneity between BAV cases with or without TAA (Supplementary Table 3) and between BAV cases with or without a positive family history of BAV and/or TAA (Supplementary Table 4), suggesting that BAV probably has an impact on the risk of TAA because of altered haemodynamic blood flow and aortopathy from different mechanisms instead of sharing molecular mechanisms with TAA that has an impact on both aorta and valve tissue^47^. In addition, we did not find evidence for heterogeneity in the association results at *GATA4* and BAV subtypes (Supplementary Table 5) and among male and female subjects (Supplementary Table 6).

### Implication of rs6601627 and rs3729856 in other CHDs

To investigate whether the two variants at *GATA4* that we report are involved in development of other and more severe CHDs, we tested for association with 806 cases of ToF along with 5,029 matched controls and performed association tests for the two variants as described previously^48^. In an additive genetic model, we did not find evidence for association between the noncoding rs6601627 and ToF (MAF=0.03, OR=0.89, 95% confidence interval (CI) 0.67–1.20, *P*=0.46); however, for *GATA4* p.Ser377Gly, the association was nominally significant (MAF=0.11, OR=1.24, 95% CI 1.06–1.45. *P*=0.007). This suggests that the regulatory variant associated with BAV may act in a highly tissue or developmentally controlled manner to cause only BAV and not other CHDs, whereas *GATA4* missense changes may have a more broad impact on other CHDs.

### Missense variant in *DHX38*

The GWAS highlighted a rare missense variant (0.14% frequency in controls) in *DHX38* (p.Thr1221Met) with a strong association with BAV (OR=13.14, 95% CI 5.39–32.04, *P*=1.5 × 10^−8^) in the discovery sample (Supplementary Fig. 2). After genotyping of this variant in 720 cases and 5,831 controls, only 22 copies of the rare allele were identified (5 in cases and 17 in controls), providing a replication *P* value of 0.05. Additional large studies will be needed to confirm this rare variant association with BAV.

### GATA4 deficiency impairs EndoMT in iPSC-derived cells

We investigated the biological impact of GATA4 in the EndoMT process required for human valve formation. Human iPSCs were generated from peripheral blood mononuclear cells of a donor with normal trileaflet aortic valve, using non-integrated DNA vectors containing *OCT4*, *SOX2*, *C-MYC* and *KLF4 (*ref. 49). The pluripotency of iPSCs was confirmed by expression of OCT4, SOX2, NANOG and SSEA4, TRA-1-60 and TRA-1-81 (Supplementary Fig. 7a,b). In addition, iPSCs generated teratoma-containing tissues from three germ layers, demonstrating their pluripotency *in vivo* (Supplementary Fig. 7c). In a previous study, wild-type GATA4 localized completely in the nucleus, whereas GATA4 mutant p.Ser377Gly (a C-terminal mutant) was shown to be partially distributed to the cytoplasm, indicating a LoF mutation^50^. To evaluate whether disruption of *GATA4* may result in a LoF phenotype, iPSCs were electrotransfected with plasmid containing Cas9, *GATA4* single guide RNA (sgRNA) and green fluorescent protein (GFP) as an indicator for transfection^51^. As control, iPSCs were transfected with plasmid containing Cas9 and GFP. Transfected cells were enriched by flow cytometry sorting based on GFP positivity (Supplementary Fig. 8a–c). iPSCs were differentiated into ECs with efficiency above 90% (Supplementary Fig. 8d).

**Figure 3:**
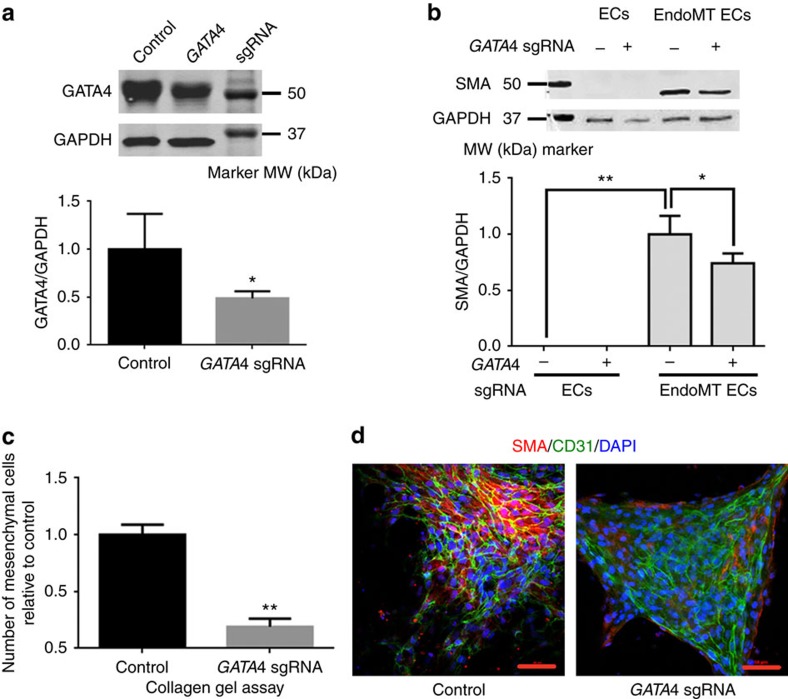
EndoMT is a key process in aortic valve development and is impaired by GATA4 deficiency. (**a**) Western blot of GATA4 and GAPDH from control and *GATA4* sgRNA ECs. *GATA4* sgRNA ECs were differentiated from iPSCs transfected with px458 with *GATA4* sgRNA and enriched by GFP. Control ECs were derived from iPSCs with px458 and enriched by GFP. An uncropped version is presented in Supplementary Fig. 9. Lower panel: quantification of western blot data. The data were normalized to control ECs. Experiments were repeated three times; averages and standard derivations were plotted. (**b**) Western blot of SMA and GAPDH from control ECs, control ECs undergoing EndoMT, *GATA4* sgRNA ECs and *GATA4* sgRNA ECs undergoing EndoMT. An uncropped version is presented in Supplementary Fig. 10. Lower panel: quantification of western blot data. The data were normalized to control ECs undergoing EndoMT. Experiments were repeated three times; averages and standard derivations were plotted. (**c**) Numbers of mesenchymal cells from control and *GATA4* sgRNA in collagen gel assay. The data were normalized to control. Experiments were repeated three times; averages and standard derivations were plotted. (**d**) Immunofluorescence staining of SMA and CD31 of the control and *GATA4* sgRNA undergoing EndoMT. Scale bars, 50 μm. EC, endothelial cell; EndoMT, endothelial-to-mesenchymal transition; iPSC, induced pluripotent stem cell; kDa, kilodalton; MW, molecular weight. *P<0.05; **P<0.01.

The GATA4 level was significantly lower in ECs from the *GATA4* sgRNA-transfected group than control (Fig. 3a), indicating successful targeting to *GATA4*. When EndoMT was induced by TGFβ2 and BMP2 in ECs, smooth muscle actin (SMA), a mesenchymal marker gene was upregulated in control cells (Fig. 3b). Noticeably, the *GATA4* sgRNA group showed significantly lower SMA levels (Fig. 3b). ECs were also explanted to collagen gel to induce EndoMT^27^. The *GATA4* sgRNA group showed significantly fewer mesenchymal cells migrating out after 3 days than control cells (Fig. 3c). Cells undergoing EndoMT express SMA and CD31 simultaneously at a certain point^27^. Immunofluoresence staining of SMA and CD31, markers of EndoMT^27^, also showed significantly less SMA and CD31 double-positive cells in the *GATA4* sgRNA group (Fig. 3d). These results indicate that EndoMT was impaired by disruption of *GATA4* with *GATA4* sgRNA.

## Discussion

In this study we find variants associated with BAV that reach genome-wide significance. We identified association with a low-frequency noncoding variant 151 kb from *GATA4*, as well as a common missense variant in *GATA4.* Although we cannot yet confirm the mechanism of action of the noncoding variant(s) on chromosome 8 on aortic valve development, chromatin conformation experiments suggest that the region near the associated variants appears to loop and physically interact with regions intronic to *GATA4*. This hypothesis could be tested in future functional experiments to investigate whether the noncoding BAV-associated variants identified here affect expression of *GATA4* at a critical time in heart development. This could possibly disrupt EndoMT, a process important for normal trileaflet aortic valve formation.

GATA4, a zinc-finger transcription factor, is one of three major transcription factors, together with Nkx2.5 and TBX5, that are critical for heart differentiation^52^. Although not previously associated with BAV, the *GATA4* gene is a plausible biological candidate. The missense *GATA4* mutation G296S disrupts the transcriptional cooperativity between GATA4 and TBX5, resulting in abnormal cellular functions related to morphogenetic defects^53^. Many mutations in *GATA4* have been previously reported to be found in different kinds of CHDs atrial septal defect^32^,^33^,^34^,^54^,^55^,^56^,^57^,^58^, ventricular septal defect^32^,^34^,^57^,^59^,^60^ and ToF^34^,^57^,^61^, although mostly tested in family studies. Furthermore, the *GATA4* mutations have been identified in CHD patients with various ancestries: European^32^,^33^,^34^,^58^, Asian^32^,^54^,^55^,^57^,^59^,^60^,^61^ and Native and Hispanic American^34^. In addition, *GATA4* knockout mice are embryonically lethal with heart defects. Mice that are missing *GATA5* also develop BAV^39^, and rare *GATA5* mutations have been identified in humans with BAV^37^.

We have provided evidence for the complexity of the BAV phenotype, with multiple genetic variants of incomplete penetrance contributing to susceptibility. To assess whether the two variants that we report are specific for BAV or whether they are also implicated in other CHDs, we studied cases of ToF, characterized by several cardiac malformations including an over-riding aorta, pulmonic stenosis, ventricular septal defect and right ventricular hypertrophy. We found that the common coding variant in *GATA4* (p.Ser377Gly) was associated with an increased risk of ToF, whereas the low-frequency noncoding variant (rs6601627) was not associated. We speculate that the low-frequency noncoding variant disrupts a regulatory element that plays a critical role in regulating *GATA4* expression in a precise time of cardiac embryogenesis that may have an impact on the valve more specifically, whereas the common *GATA4* missense variant might disrupt GATA4 function more generally and increase the risk of several cardiac malformations, including ToF. The frequencies of the associated variants at the *GATA4* locus (variants with *r*^2^>0.6 in EUR samples of 1000 G) vary among different populations^62^. For example, among the noncoding variant rs6601627 and its 115 correlated variants, 108 variants have MAF<0.01 and 73 are monomorphic in East Asians^62^. Association studies in other populations will be critical for determining whether the association exists in other populations and may be helpful at narrowing the associated interval.

To investigate the possible role of GATA4 in aortic valve development, we used sgRNA-guided Cas9 to disrupt *GATA4* in iPSCs from a healthy human donor with normal tricuspid aortic valves (TAVs). The iPSCs were differentiated into ECs and then induced to mesenchymal cells through EndoMT. We demonstrated that deficiency of GATA4 impaired the EndoMTs, a critical step in valve formation^16^ (Fig. 3). This indicates that GATA4 is required for aortic valve formation and that disruption of the *GATA4* gene, either by noncoding or protein-altering variants, may affect aortic valve formation.

## Methods

### GWAS genotyping and genotype imputation

We performed genotyping of a combined set of 498,075 GWAS variants, including 217,957 protein-altering variants, using a GWAS+exome chip array (Illumina Human CoreExome). To avoid any potential batch effects, cases and controls were genotyped using the same array in the same genotyping centre (Sequencing and Genotyping Core at the University of Michigan). Genotype calling was performed using GenTrain version 2.0 in GenomeStudio V2011.1 (Illumina) using identical cluster files for cases and controls. Samples with <98% genotype calls, evidence of gender discrepancy, duplicates as well as individuals with non-European ancestry identified by plotting the first 10 genotype-driven principal components were excluded from further analysis. We performed variant-level quality control (QC) by excluding 22,983 variants that met any of the following criteria: variants with a cluster separation score<0.3, <98% genotype call rate or deviation from Hardy–Weinberg equilibrium (*P*<1 × 10^−5^). We phased the autosomal genotype data using SHAPEIT2 (ref. 63) and imputed variants from the HRC v1 reference panel^17^ using minimac3 (ref. 64). We excluded poorly imputed variants with imputation *r*^2^<0.3, and then merged the genotyped variants and the successfully imputed variants to a combined data set, which contains 12,320,487 variants in total.

### Description of cases in discovery cohort

We collected DNA from consented individuals with BAV from the FCVC at the University of Michigan as part of the University of Michigan BAV registry or the Cardiovascular Health Improvement Project (CHIP). All repository projects utilized for this study are approved by the University of Michigan, Medical School, Institutional Review Board (IRBMED), and informed consent was obtained from study participants. Patients were typically seen in clinic for aortic valve replacement or aortic aneurysm. Diagnoses of BAV were made by cardiac surgeons upon visual inspection of the aortic valve during open surgery for aneurysm repair or valve replacement. BAV cases with major syndromic connective-tissue disorders (for example, Marfan syndrome) were excluded. DNA was isolated from peripheral blood lymphocytes.

### Description and selection of controls in discovery cohort

We identified potential controls from a surgical-based biobank, the Michigan Genomics Initiative (MGI), that were genotyped with the same GWAS array (Illumina Human CoreExome). After excluding those with possible aortic disease (*n*=1,586, Supplementary Table 7), we were left with 15,642 potential controls with GWAS data. We performed age-matching by requiring controls to have a birth year within −5 and +10 years of the case. From the available controls in the appropriate age and sex category, we selected the best ethnic match for each case and repeated the greedy algorithm until a control was selected for each case. We repeated the entire process so that 10 controls were selected for each case. We opted for this approach to provide the best ancestry-matching between cases and controls, to reduce the potential for false-positives due to ethnicity mismatch and to also provide the most power for rare variants that increase the risk of BAV by including the highest number of matching controls. All MGI research subjects provided informed consent.

### Statistical analyses

In the discovery cohort, we performed association testing for the BAV status using logistic regression with single genetic variants (295,759 with MAF>1%), with age, sex and the first four principal components as covariates using PLINK for hard call genotypes^65^ and the EPACTS software (http://csg.sph.umich.edu//kang/epacts/) for imputed dosages. We identified two genetic variants that were directly genotyped and reached genome-wide significance levels (*P*<5 × 10^−8^). We observed no evidence for inflation because of population stratification (*λ*=1.033, Supplementary Fig. 1). We observed a genotyped missense variant within 200 kb (rs3729856) of one of the significant noncoding variants (rs6601627) and selected this third variant for follow-up in additional samples.

### Association with ToF

A total of 835 unrelated ToF cases and 5,159 controls were genotyped and imputed from 1,000 Genomes Phase 3 for the region 11–12 MB on chromosome 8 using IMPUTE2 (refs 48, 66). The association tests were performed using logistic regression of the ‘best-guess' genotypes for all imputed single-nucleotide polymorphisms (SNPs) with IMPUTE2 info score ≥0.5 and with MAF ≥0.01 in controls using SNPTEST^67^. This study has been approved by Newcastle and North Tyneside NHS Research Ethics Committee.

### Incorporating gene expression and epigenetics data

We assessed expression levels of relevant genes using the GTEx server (http://www.gtexportal.org/home/)^23^. We obtained the Hi-C interaction calls from Rao *et al*.^45^,^46^ and ChIA-PET interactions from Phanstiel *et al*.^45^,^46^ available through the ENCODE DCC accessions ENCSR000FDB and ENCSR752QCX (https://www.encodeproject.org/). ChromHMM data are displayed from the K562 Genome Segmentation by ChromHMM from ENCODE/Analysis available at https://genome.ucsc.edu/ and is created through chromatin segmentation using eight histone modifications, CTCF, Pol2 and open chromatin annotations^68^.

### IPSC generation and culture

The procedure of iPSC derivation was performed according to methods we described^49^: Peripheral blood mononuclear cells (PBMC) were separated from human peripheral blood with lymphocyte seperation medium (LSM) (MP Biomedicals LLC.), cultured in medium containing Iscove's modified Dulbecco's medium (IMDM) (Life Technologies Corp.), 10% fetal bovine serum (Life technologies Corp.), thrombopoietin (TPO), stem-cell factor (SCF) and FMS-like tyrosine kinase-3 (FLT-3) at a final concentration of 100 ng ml^−1^, granulocyte–macrophage colony-stimulating factor and interleukin-3 at a final concentration of 10ng ml^−1^ (Peprotech Inc.) and penicillin–streptomycin (Life Technologies Corp.), and electrotransfected with episomal DNA plasmids containing *OCT4, SOX2, KLF4* and *C-MYC* using Nucleofector 2 Device (Lonza Corp.). At around day 30 post infection, the colonies became compact. The colonies were mechanically picked up from the culture dishes and first cultured with mouse embryonic fibroblasts for three passages^69^ and transited to TesRE8 medium (Stemcells Inc.) on matrigel-coated (BD Corp.) dishes. iPSCs were passaged every 4–6 days with Versene (Life Technologies Corp.). In addition, iPSCs from passages 25–35 were used in experiments.

### Teratoma formation in immune-deficient mice

Conduction of Animal experiments was in compliance with the regulations of the Unit for Laboratory Animal Medicine at the University of Michigan. Two million iPSCs were injected subcutaneously into each flank of the recipient male, 6–8-week-old nonobese diabetic-severe combined immunodeficient mice (Jackson Laboratory, Bar Harbor, Maine). Three to five weeks after injection, teratomas were collected from the mouse flanks and fixed with formalin (Thermo Corp.) for 2 days. The tumours were then embedded in paraffin, and sections were prepared with a microtome (Leica Corp.) and stained with haematoxylin and eosin staining solutions from Thermo Corp. The slides were examined and micrographs were taken under brightfield with microscope (Nikon Corp.).

### *GATA4* sgRNA design and electrotransfection of iPSCs

sgRNAs were designed to target *GATA4* exon2 (the first coding exon) with sgRNA design tool (http://www.genome-engineering.org) developed by Zhang and co-workers^51^. Sequence of *GATA4* sgRNA was 5′-CGCGCCGTGCATGAAGGCGCCGG-3′. Target site was chr8:-11565888. Quality score was 93. Minimal number of mismatch nucleotides in offsite targets was 3. SgRNAs were cloned into PX458, which contains SpCas9-2A-EGFP, using AgeI and EcoRI at 5′ and 3′ cloning sites^51^. One million iPSCs were electrotransfected with constructed 5 μg PX458 containing *GATA4* sgRNA, using the Lonza Human Stem Cell Nucleofector Kit 2 with programme U-023 on Nuclefector 2 device (Lonza Ltd.). Another one million iPSCs were electrotransfected with the PX458 vector as control under the same conditions.

### EC differentiation from iPSCs

To differentiation iPSCs into ECs, iPSCs were dissociated with Versene (Life Technologies Corp.) into single cells and seeded at 2 × 10^4^ cells per cm^2^ with the TesRE8 (Stemcell Technology Inc.) medium supplemented with Rocki (Y27632, Stemgent Inc.). When the cells reached a confluence of 20–30%, the medium was changed into a differentiation medium, which contained DMEM-F12 (Life Technologies Corp.), B27 supplement without vitamin A (Life Technologies Corp.), L-glutamine (Life Technologies Corp.), penicillin–streptomycin (Life Technologies Corp.), 400 μM 1-thioglycerol (Sigma Corp.), 50 μg ml^−1^ ascorbic acid (Sigma Corp.), 25 ng ml^−1^ BMP4 (R&D Systems Corp.) and 6 μM GSK3 inhibitor CHIR99021 (Sigma Corp.). Differentiation medium was refreshed daily for 3 days. Then, cells were dissociated with Accutase (Life Technologies Corp.) and seeded at 1 × 10^4^ cells per cm^2^ on matrigel (BD Corp.)-coated dishes with an EC medium containing Stempro34(Life Technologies Corp.), Stempro34 supplement (Life Technologies Corp.), L-glutamine (Life Technologies Corp.), penicillin–streptomycin (Life Technologies Corp.) and 50 ng ml^−1^ vascular endothelial growth factor (Peprotech Inc.). Medium was refreshed every 2 days for 13 days.

### Immunofluorescence staining and flow cytometry

Immunofluorescence staining and flow cytometry were performed as follows: first, cells were fixed in 4% formaldehyde (Thermo Corp.) for 1 h at room temperature, and then the cells were washed with DPBS (Thermo Corp.) once and incubated with primary antibodies for 2 h at room temperature^70^. The following primary antibodies were used: anti-OCT4 (mouse IgG, dilute 500 times upon usage, sc-5,279, Santa Cruz Biotechnology Inc.), anti-SOX2 (mouse IgG, dilute 500 times upon usage, sc-365964, Santa Cruz Biotechnology Inc.), anti-NANOG (rabbit polyclonal, dilute 500 times upon usage, REC-RCAB004PF, Cosmo Inc.), anti-SSEA4 (mouse IgG, dilute 100 times upon usage, 60062, Stemcell Technology Inc.), anti-TRA-1-60 (mouse IgM, dilute 100 times upon usage, 60,064, Stemcell Technology Inc.), anti-TRA-1-81 (mouse IgM, dilute 100 times upon usage, 60,065, Stemcell Technology Inc.), anti-CD31 (rabbit polyclonal, dilute 500 times upon usage, ab28364, Abcam Inc.) and anti-SMA (mouse IgG, dilute 1,000 times upon usage, A5228, Sigma Corp.). Cells were washed three times with DPBS (Thermo Corp.), and then incubated with secondary antibodies for 1 h at room temperature. The following fluorochrome-conjugated secondary antibodies were used: Alexa Fluor 488 goat anti-rabbit IgG (goat, dilute 1,000 times upon usage, A11034, Thermo Corp.), Alexa Fluor 488 goat anti-mouse IgG (goat, dilute 1,000 times upon usage, A32723, Thermo Corp.) and Alexa Fluor 594 goat anti-mouse IgG (goat, dilute 1,000 times upon usage, A11032, Thermo Corp.). Slides were mounted with anti-fade mounting media containing 4,6-diamidino-2-phenylindole (Prolong gold, Life Technologies Corp.), and were observed on a Nikon A1 confocal microscope (Nikon Corp.). In a flow cytometry study, electrotransfected iPSCs were dissociated into single cells with Accutase (Stemcell Technology Inc.), and applied to the MoFlo Astrios (Beckman Coulter Inc.) flow cytometry machine.

### Western blot analysis

Whole-cell extracts were prepared using RIPA buffer (1% NP-40, 1% sodium deoxycholate, 0.1% SDS, 0.15 M NaCl, 0.01 M sodium phosphate, 2 mM EDTA, 50 mM sodium fluoride, 0.2 mM Na_3_VO_4_.2H_2_O, 100 U ml^−1^ protease inhibitor), resolved on SDS–PAGE gels and transferred to acetate cellulose membranes. Primary antibodies used were anti-GATA4 (rabbit IgG, diluted 500 times upon usage, 36,968, Cell Signaling Technology Inc.), anti-SMA (mouse IgG, diluted 300 times upon usage, A5228 Sigma) and anti-GAPDH (rabbit IgG, diluted 2,000 times upon usage, sc25778, Santa Cruz Inc.). Secondary antibodies used were IRDye800CW Donkey anti-Mouse (92532212), IRDye680LT Donkey anti-Rabbit (92568023), IRDye800CW Donkey anti-Rabbit (92532213; all secondary antibodies were diluted 500 times upon usage and purchased from Licor Inc.). The Licor western blot detection system was used for the dual-colour imaging. Uncropped versions of western blots are presented in Supplementary Figs 9 and 10. ImageJ was used for quantification of bands. Each band was normalized by GAPDH. Experiments were repeated three times. Average value and s.d. were plotted.

### Endothelial-to-mesenchymal transition and collagen gel assay

EndoMT was induced by changing medium to an EndoMT-inducing medium that contained stempro34 medium with stempro34 supplement (Life Science Technology Corp.), L-glutamine (Life Technologies Corp.), penicillin–streptomycin (Life Technologies Corp.), 200 ng ml^−1^ BMP2 (Peprotech Inc.) and 50 ng ml^−1^ TGFβ2 (Peprotech Inc.). Non-EndoMT control groups were kept in EC medium containing Stempro34 (Life Technologies Corp.), Stempro34 supplement (Life Technologies Corp.), L-glutamine (Life Technologies Corp.), penicillin–streptomycin (Life Technologies Corp.) and 50 ng ml^−1^ vascular endothelial growth factor (Peprotech Inc.). Cells were collected 3 days after induction.

Type I collagen (Sigma Corp.) at 1 mg ml^−1^ (final concentration) was mixed with stempro34 medium, stempro34 supplement (Life Science Technology Corp.) and 50 mM NaOH (Sigma Corp.). The mixture was poured into 24-well tissue culture plates (0.5 ml per well) and allowed to gel in 5% CO_2_ incubator at 37 °C for 30 min. And then 0.5 ml EndoMT-inducing medium was added. After 3 days, pictures of cells were taken with 100 times magnificance under the Eclipse Ti-U inverted research microscope (Nikon Corp.). Mesenchymal cells that migrated out in three pictures from different fields were counted. Experiments were repeated three times. Average value and standard derivation were plotted.

### Genetic association replication cohorts

*CHIP.* In the CHIP replication cohort, an additional 140 BAV cases from the University of Michigan FCVC biobank were collected. These samples were genotyped using the same GWAS array as the discovery cohort, but were only examined for the three variants described here. The association was tested using PLINK^65^ with 1,400 age-, sex- and ancestry-matched controls from the MGI study, which were independent samples from previously used controls. Informed consent was obtained from all participants and approval was obtained from the Institutional Review Board of the University of Michigan Medical School.

*Montreal Heart Institute.* In the Montreal Heart Institute (MHI) biobank, 305 BAV cases and 2,746 controls were collected and genotyped on the Illumina Core Exome array at the MHI Pharmacogenomic Centre. Controls were selected by excluding those with myocardial infarction (MI), percutaneous coronary intervention (PCI), Angina, congestive heart failure (CHF), valve defects, heart surgeries, heart arrest, atrial fibrillation and sudden cardiac death. Genotyping was performed with the Illumina HumanExome array. Association analysis was performed in PLINK^65^ using a logistic regression model correcting for sex, age and principal components of ancestry 1–10. The project has been approved by the Ethics Committee of the MHI and informed consent was obtained from study participants.

*Partners HealthCare.* In the Partners HealthCare cohort, 452 Caucasian BAV cases were identified from the electronic medical records (EMRs) of Partners HealthCare (Boston, MA). Individual echocardiographic images were reviewed to confirm BAV diagnosis. Whole-blood DNA was genotyped using the Illumina Omni2.5 Beadchip. The Framingham Heart Study dbGaP cohort, genotyped using the Illumina Omni5.0 Beadchip, was used as controls. QC and population stratification of the genotype data were performed in PLINK^65^. SNPs with MAF less than 1%, without physical map reference, not in Hardy–Weinberg equilibrium (*P*<10^−4^), differential missingness (*P*<10^−5^), were removed. Related individuals (PI_HAT>0.25) were excluded. Genome-wide IBD and IBS were used to detect outliers and clusters. After merging case and control genotypes, additional genotypes have been imputed against the 1,000 Genome reference (phase3) and HRC (Michigan University) panels using SHAPEIT2 (ref. 63) and IMPUTE2 (ref. 66). After QC, 452 cases and 1,634 controls (1,094 males+992 females) with 7.5 million markers were analysed using an additive logistic regression model accounting for gender, age and principal components. This study has been approved by Partner's HealthCare Human Research Committee, and informed consent was obtained from study participants.

*University of Texas Health Science Centre.* In the University of Texas Health Science Centre cohort, 765 patients with sporadic TAAs or aortic dissections were collected and genotyped. In all, 874 genotypes from dbGAP (NINDS Neurologically Normal control collection) were used as controls. QC and population stratification of the genotype data were performed in PLINK^65^. SNPs with MAF less than 1% or missing more than 1% of genotypes were excluded. Multidimensional scaling was used to detect and exclude population outliers. We imputed additional genotypes against 1,000 Genomes Phase3 using SHAPEIT2 (ref. 63) and IMPUTE2 (ref. 66). After QC, a total of 152 BAV cases and 633 TAV cases or 874 controls were analysed using an additive logistic regression model accounting for gender and principal components. This study has been approved by the Committee for the Protection of Human Subjects at UT Health Science Center at Houston, and informed consent was obtained from study participants.

*ASAP–ARTIST–POLCA–Olivia cohort.* Three cohorts ASAP, ARTIST and POLCA were included in this replication group with a total of 275 BAV cases and 1,686 controls used for analysis. The POLCA/Olivia cohort is a merged cohort with a total of 1,295 individuals. The ASAP cohort consists of 429 patients genotyped on Illumina 610wQuad beadchips. Approximately 588,400 SNPs were provided after QC. The Artist cohort consists of 406 samples genotyped with Omni2.5 Quad beadchips on 2,443,180 SNPs. In POLCA, 625 control samples were genotyped on Illumina 610kwQuad, and in Olivia 670 control samples were genotyped on Illumina 1M-genotyping arrays. The vast majority of included samples are of Scandinavian ancestry. For the ASAP database, where ancestry is specifically registered, this corresponds to >95% of the individuals, supported by PCA plots of genotype clustering. Imputation was performed using Impute2 from 1,000G phase1 v3 (ref. 66). Analysis was performed using SNPTEST^67^, with age, sex and first 10 principal components as covariates. This study was approved by the Regional Ethical Committee of Stockholm, and informed consent was obtained from study participants.

*BioMe.* The Mount Sinai Bio*Me* Biobank (Bio*Me*) is an ongoing, prospective, hospital- and outpatient-based population research programme operated by The Charles Bronfman Institute for Personalized Medicine at Mount Sinai and has enroled over 33,000 participants since September 2007. Bio*Me* is an EMR-linked biobank that integrates research data and clinical care information for consented patients at The Mount Sinai Medical Center, which serves diverse local communities of upper Manhattan with broad health disparities. Bio*Me* populations include 25% of African ancestry (AA), 36% of Hispanic Latino ancestry (HL), 30% of white European ancestry (EA) and 9% of other ancestry. The Bio*Me* disease burden is reflective of health disparities in the local communities. Bio*Me* operations are fully integrated in clinical care processes, including direct recruitment from clinical sites waiting areas and phlebotomy stations by dedicated recruiters independent of clinical care providers, prior to or following a clinician standard of care visit. Recruitment currently occurs at a broad spectrum of over 30 clinical care sites. Information on BAV status, age and sex was derived from participants' EMRs. BAV cases were defined as Bio*Me* participants with the ICD-9 code 746.4 (Congenital insufficiency of aortic valve). In total, there were 41 BAV cases with available genotyping data (8 AA and 13 HL BAV cases genotyped on the Infinium Multi-Ethnic Global BeadChip from Illumina as well as 13 additional HL and 7 EA BAV cases genotyped on the Illumina HumanOmniExpressExome-8 v1.0 BeadChip. For each case, three controls were selected by genetically matching using the first two genetic principal components and stratification by age and sex. Logistic regression was performed in PLINK for the three SNPs in the four groups^65^. We performed analyses both including and excluding the BioME non-European samples, and results were highly similar. We present results in this study excluding the non-European samples since there were few cases. This study has been approved by Icahn School of Medicine IRB and informed consent was obtained from study participants.

### Data availability

The data that support the findings of this study are available from the corresponding author upon reasonable request.

## Additional information

**How to cite this article:** Yang, B. *et al*. Protein-altering and regulatory genetic variants near GATA4 implicated in bicuspid aortic valve. *Nat. Commun.* **8,** 15481 doi: 10.1038/ncomms15481 (2017).

**Publisher's note:** Springer Nature remains neutral with regard to jurisdictional claims in published maps and institutional affiliations.

## Supplementary Material

Supplementary Information Supplementary figures, supplementary tables and supplementary references.

## Figures and Tables

**Table 1:**
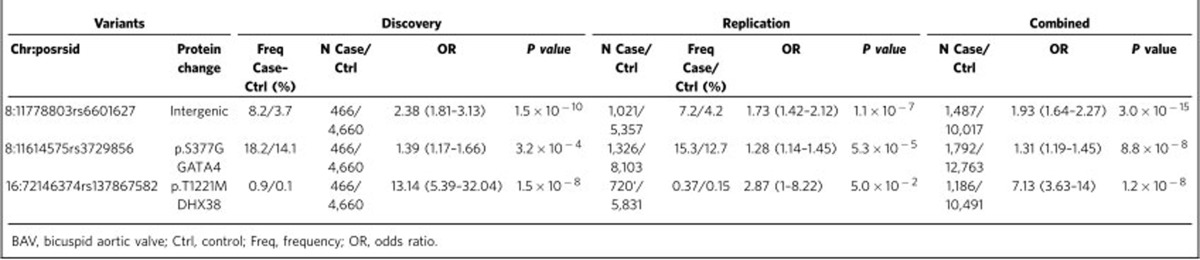
Genetic variants associated with BAV.
