# Supplementary material for: Protein-altering and regulatory genetic variants near GATA4 implicated in bicuspid aortic valve
Source: Nat Commun. 2017 May 25;8:15481. doi: 10.1038/ncomms15481 (PMC5458508; doi:10.1038/ncomms15481)
Supplement: Supplementary Information — Supplementary figures, supplementary tables and supplementary references. [file ncomms15481-s1.pdf]

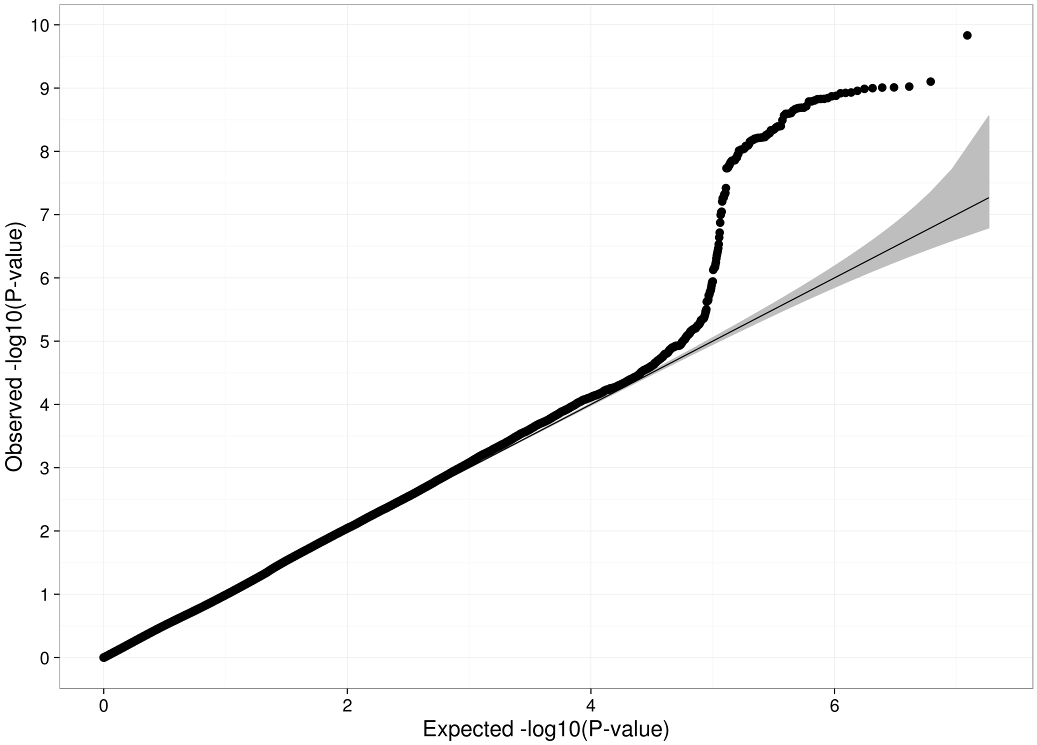

SUPPLEMENTARY FIGURE 1. Quantile-quantile plot for single-variant analysis results of BAV in the discovery cohort. Variants in this plot include all those directly genotyped using the chip array and those successfully imputed from Haplotype Reference Consortium (HRC)[1]

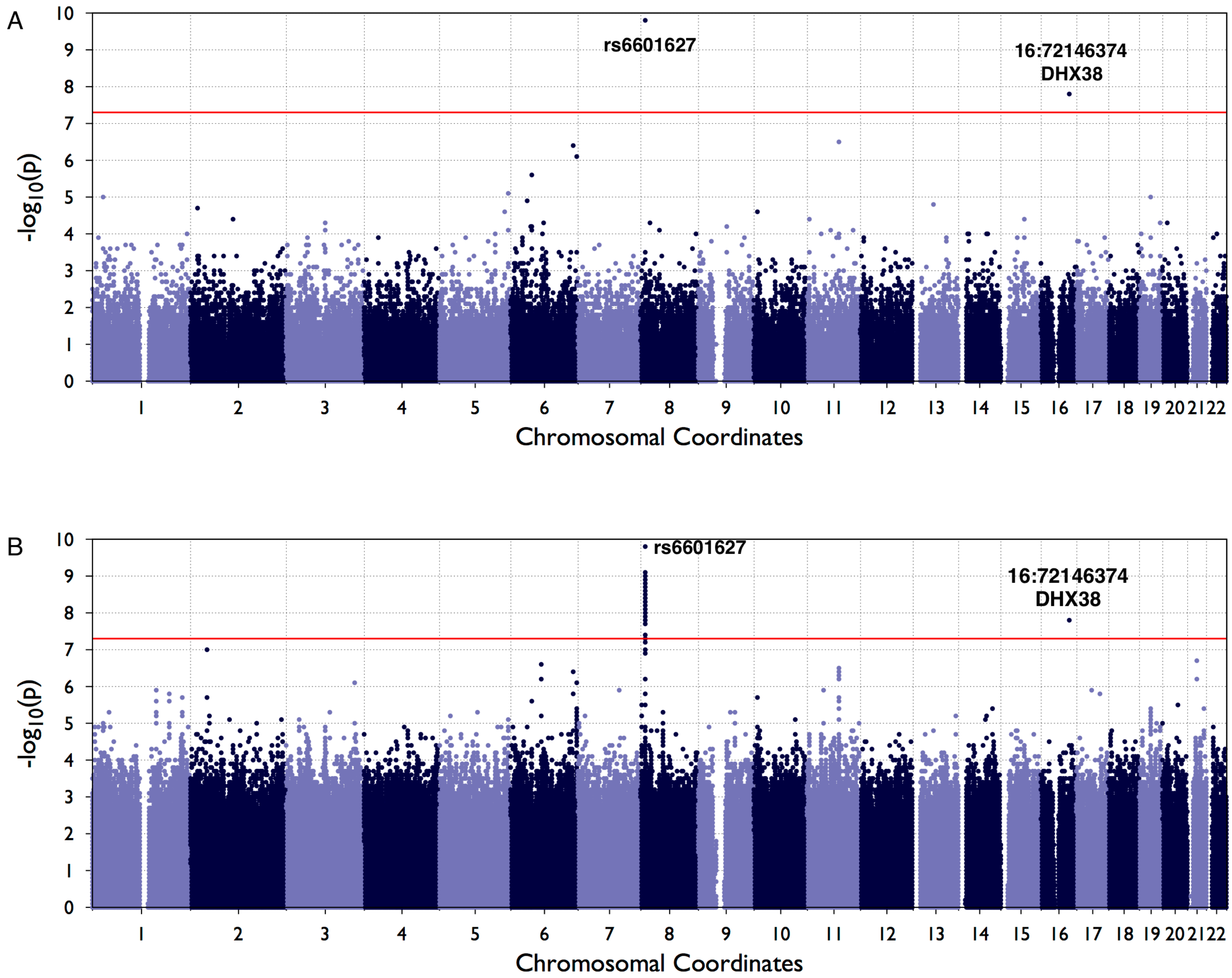

SUPPLEMENTARY FIGURE 2. Manhattan plots for single variant association tests with BAV in the discovery cohort. The red line indicates the genome-wide significance threshold ($P = 5x10^{-8}$).

(A) Before genotype imputation

After genotype imputation from the HRC[1]

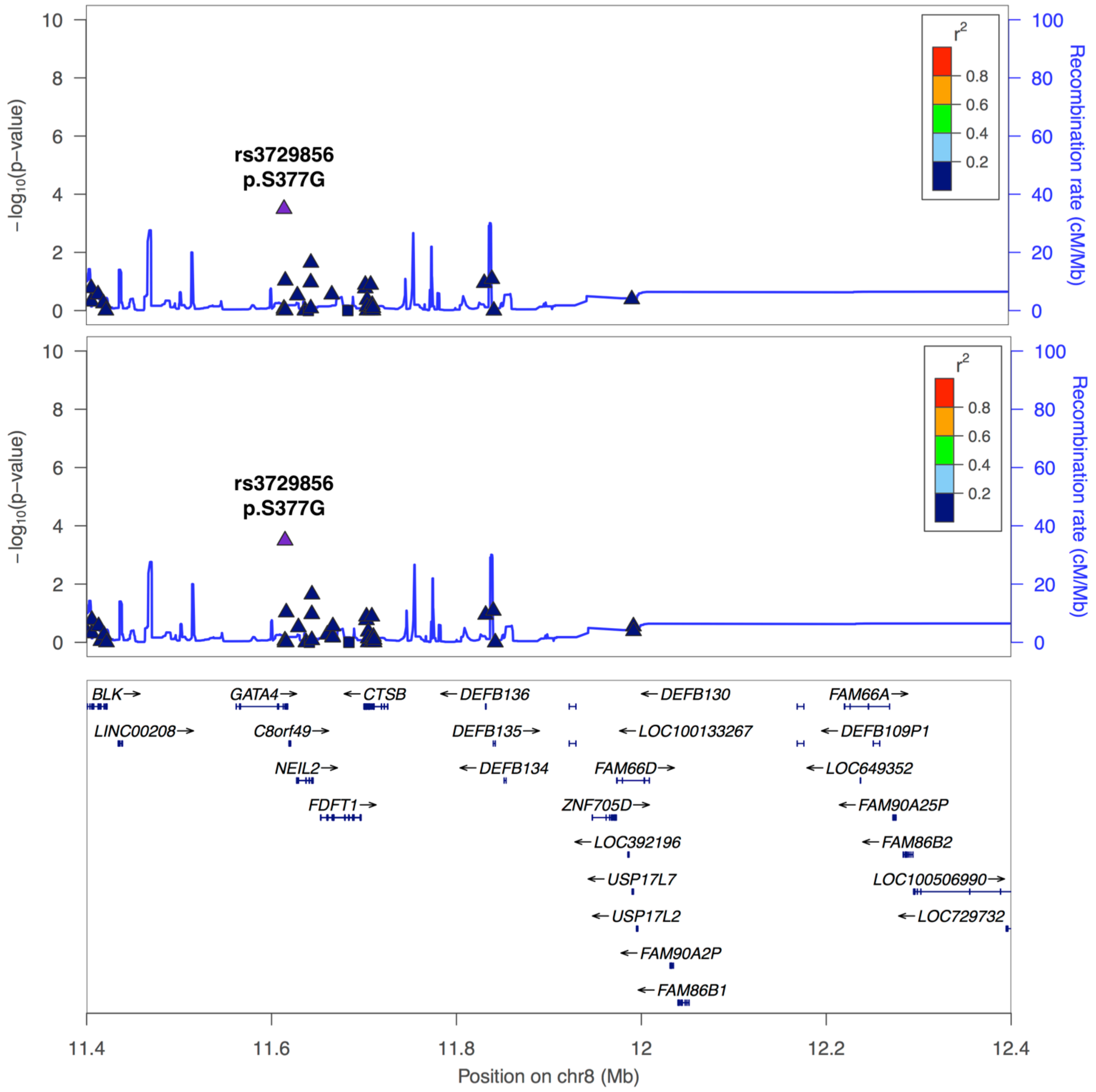

SUPPLEMENTARY FIGURE 3. Regional association plot for all coding variants of the chr8 association region near *GATA4*, as observed in the discovery cohort (N=466 BAV cases, 4,660 controls). The upper panel shows all 59 coding variants that were directly genotyped in the chip array in this region. A missense variant rs3729856 within *GATA4* was observed with p = 3.2×10^{-4}, that reached P = 8.8×10^{-8} following replication. The bottom panel includes additional 11 coding variants whose genotypes are imputed to the HRC reference[1]. Coding variants observed in this region contain missense variants (represented by triangles) and stop gain variants (represented by squares).

rs6601627

	OR	95% CI	Case/Control
**Discovery Study**			
CHIP	2.38	1.81-3.13	466/4,660
**Replication Study**			
CHIP replication	2.27	1.42-3.63	140/1,400
Partners HealthCare	1.77	1.32-2.37	452/1,634
UTHSC _BCM	1.49	0.68-3.27	62/337
UTHSC _UT	1.35	0.56-3.26	85/279
ASAP-Artist-Polca-Olivia	1.4	0.88-2.22	275/1,686
BioMe-EA-Omni	1.44	0.28-7.28	7/21
**Combined**	**1.93**	**1.64-2.27**	**1,487/10,017**

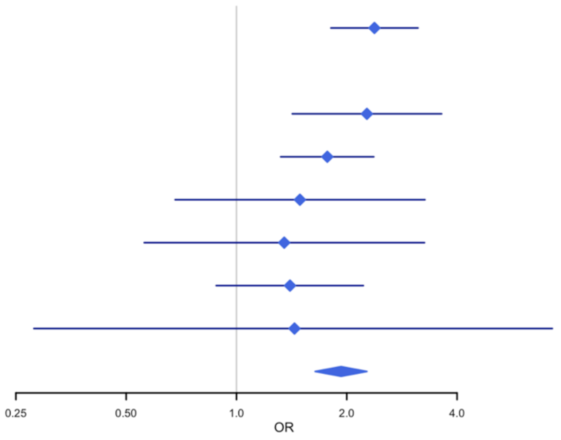

rs3729856

	OR	95% CI	Case/Control
**Discovery Study**			
CHIP	1.39	1.17-1.66	466/4,660
**Replication Study**			
CHIP replication	1.05	0.74-1.49	140/1,400
MHI	1.42	1.11-1.81	305/2,746
Partners HealthCare	1.28	1.03-1.59	452/1,634
UTHSC _BCM	1.55	0.88-2.72	62/337
UTHSC _UT	1.4	0.81-2.42	85/279
ASAP-Artist-Polca-Olivia	1.18	0.91-1.53	275/1,686
BioMe-EA-Omni	1.36	0.40-4.58	7/21
**Combined**	**1.31**	**1.19-1.45**	**1,792/12,763**

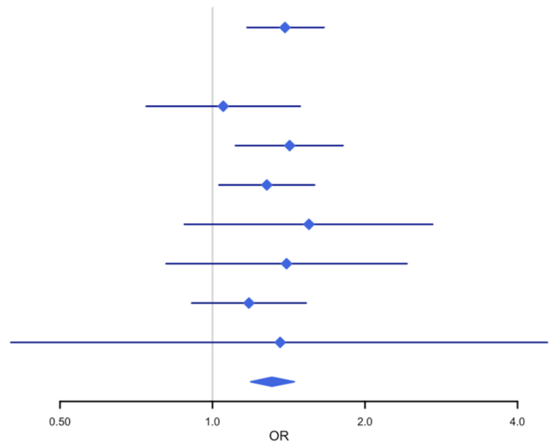

rs137867582

	OR	95% CI	Case/Control
**Discovery Study**			
CHIP	13.14	5.39-32.04	466/4,660
**Replication Study**			
CHIP replication	11.96	1.64-87.25	140/1,399
MHI	2.02	0.54-7.53	305/2,746
ASAP-Artist-Polca-Olivia	0.50	0.02-14.2	275/1,686
**Combined**	**7.13**	**3.63-14**	**1,186/10,491**

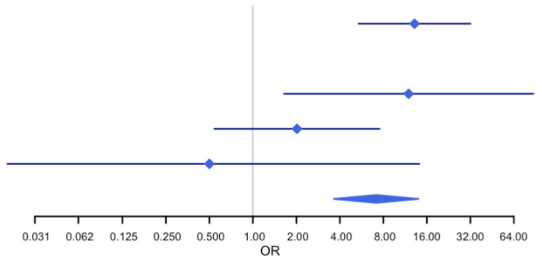

SUPPLEMENTARY FIGURE 4. Forest plots of the BAV hits near *GATA4* by stage and study. The combined results are for the meta-analysis of the discovery study and all replication studies.

rs3729856

	OR	95% CI	Case/Control
**Discovery Study**			
CHIP	1.27	1.05-1.53	466/4,660
**Replication Study**			
CHIP replication	0.95	0.66-1.37	140/1,400
Partners HealthCare	1.18	0.94-1.48	452/1,634
UTHSC _BCM	1.56	0.90-2.70	62/337
UTHSC _UT	1.45	0.86-2.45	85/279
ASAP-Artist-Polca-Olivia	1.04	0.74-1.24	275/1,686
**Combined**	**1.11**	**1.03-1.20**	**1,480/9,996**

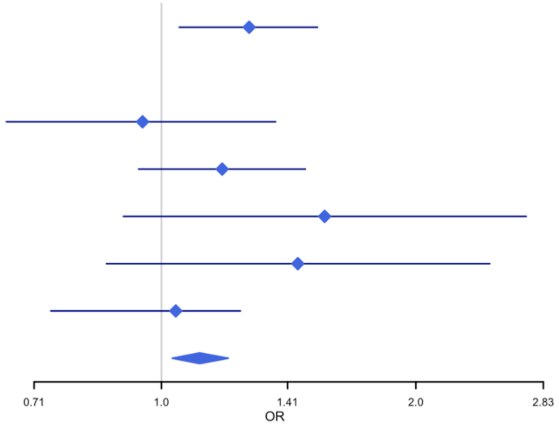

rs6601627

	OR	95% CI	Case/Control
**Discovery Study**			
CHIP	2.217	1.69-2.91	466/4,660
**Replication Study**			
CHIP replication	2.3	1.42-3.74	140/1,400
Partners HealthCare	1.68	1.24-2.27	452/1,634
UTHSC _BCM	1.65	0.73-3.73	62/337
UTHSC _UT	1.4	0.60-3.26	85/279
ASAP-Artist-Polca-Olivia	0.91	0.56-1.4	275/1,686
**Combined**	**1.4**	**1.26-1.56**	**1,480/9,996**

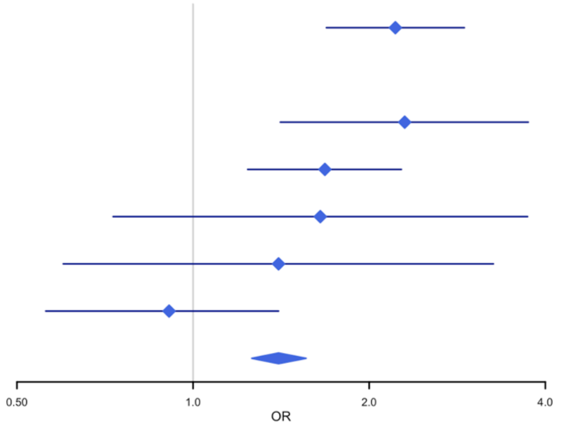

SUPPLEMENTARY FIGURE 5. Forest plots of the reciprocal conditional analysis of the two BAV hits near *GATA4* by stage and study. The combined results are for the meta-analysis of the discovery study and all replication studies.

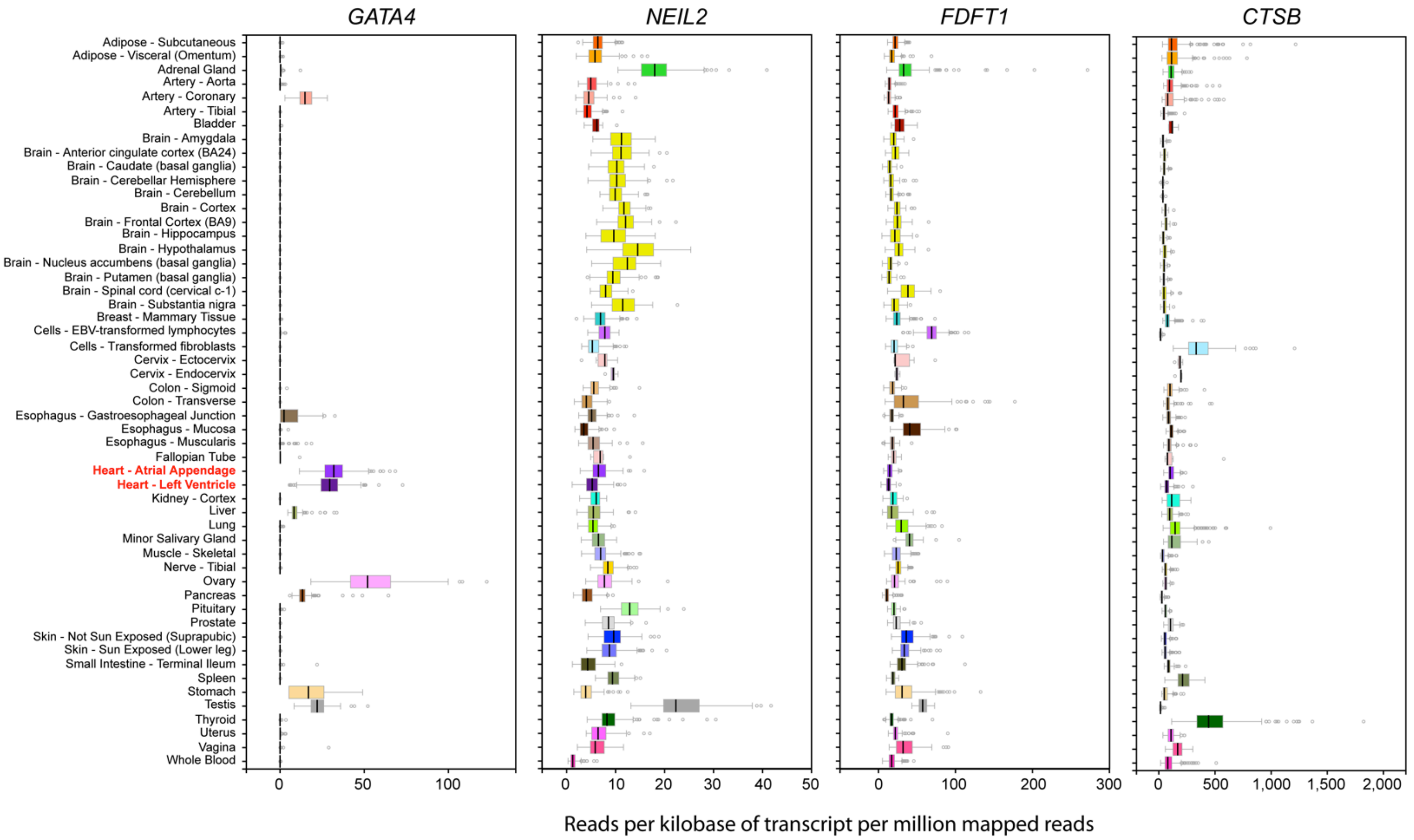

SUPPLEMENTARY FIGURE 6. The mRNA expression levels of genes surrounding the non-coding associated variant rs6601627 from the GTEx portal[2].

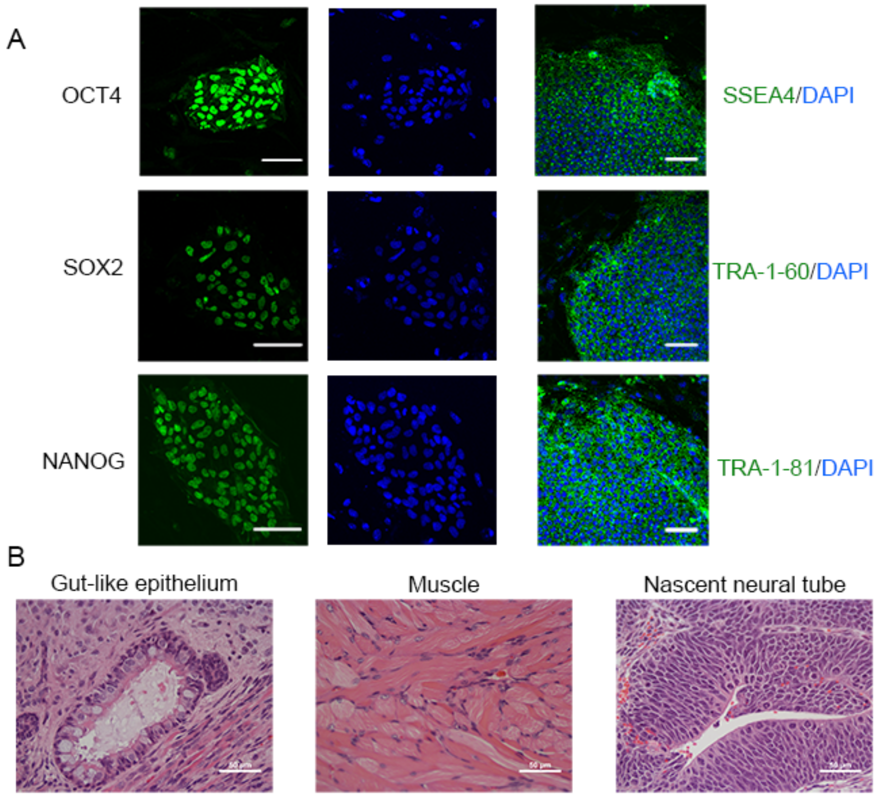

SUPPLEMENTARY FIGURE 7. iPSCs from Control patient are pluripotent.

(A) Immunofluorescence staining of OCT4, SOX2, NANOG of the iPSC colonies. The scale bars represent 50µm.

(B) Immunofluorescence staining of SSEA4, TRA-1-60 and TRA-1-81 of the iPSC colonies. The scale bars represent 50µm.

(C) H&E staining of teratomas. The scale bars represent 50µm. DAPI marks the nucleus. Abbreviations: iPSCs: induced pluripotent stem cells.

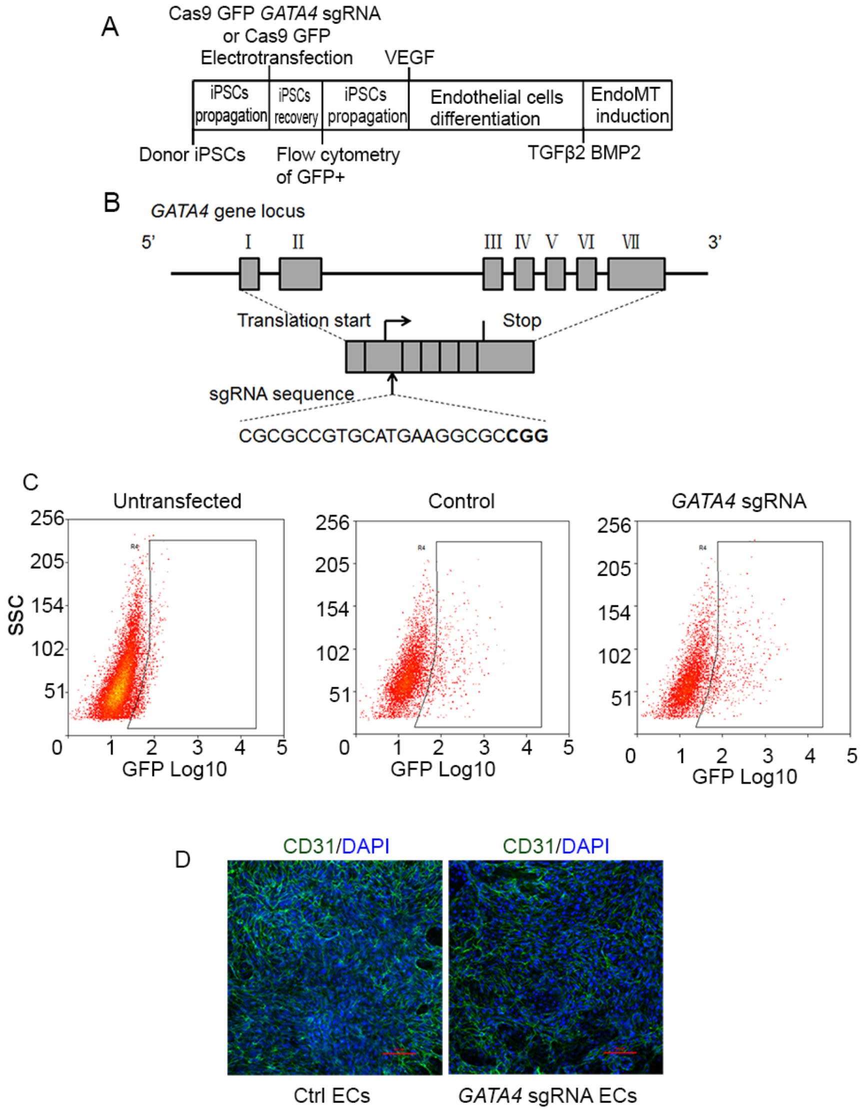

SUPPLEMENTARY FIGURE 8. *GATA4* sgRNA/cas9 electrotransfection of iPSCs.

(A) Diagram of experimental process.

(B) Illustration of *GATA4* sgRNA target site.

(C) Flow cytometry of electrotransfected iPSCs. IPSCs in "Control" group were transfected with PX458 plasmids containing Cas9 and GFP. IPSCs in *GATA4* sgRNA group were transfected with PX458 plasmids containing Cas9, GFP and *GATA4* sgRNA. Successfully transfected cells were GFP positive. GFP positive cells within the inside area were selected for further experiments.

(D) Immunofluorescence staining of CD31 on ECs differentiated from iPSCs. DAPI marks the nucleus. Scale bars represent 100µm. Abbreviations: iPSCs: induced pluripotent stem cells. EC: endothelial cells. GFP: green fluorescent protein. EndoMT: endothelial-to-mesenchymal transition.

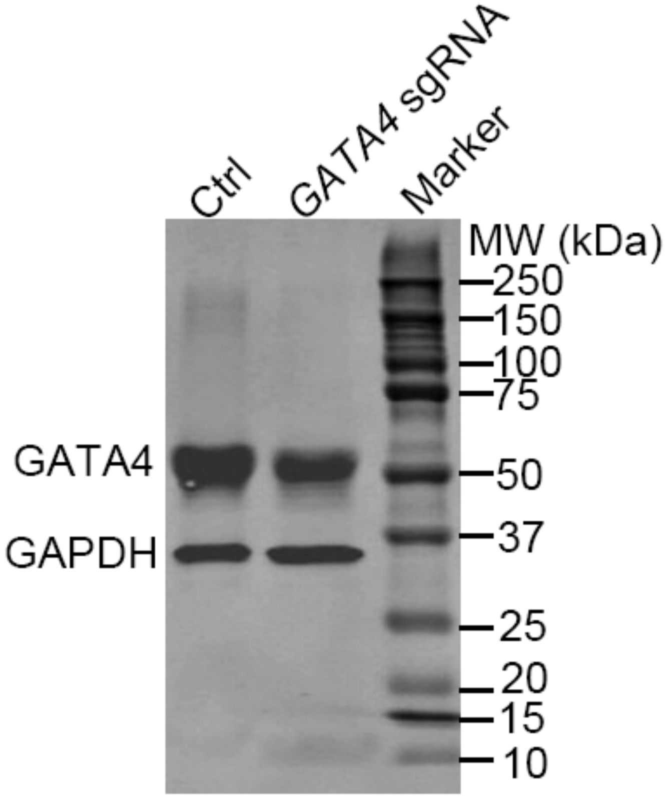

SUPPLEMENTARY FIGURE 9. Uncropped version of GATA4 and GAPDH western blot. This is uncropped version of Figure 3A. Abbreviations: Ctrl: control. MW: molecular weight. kDa: kilodalton.

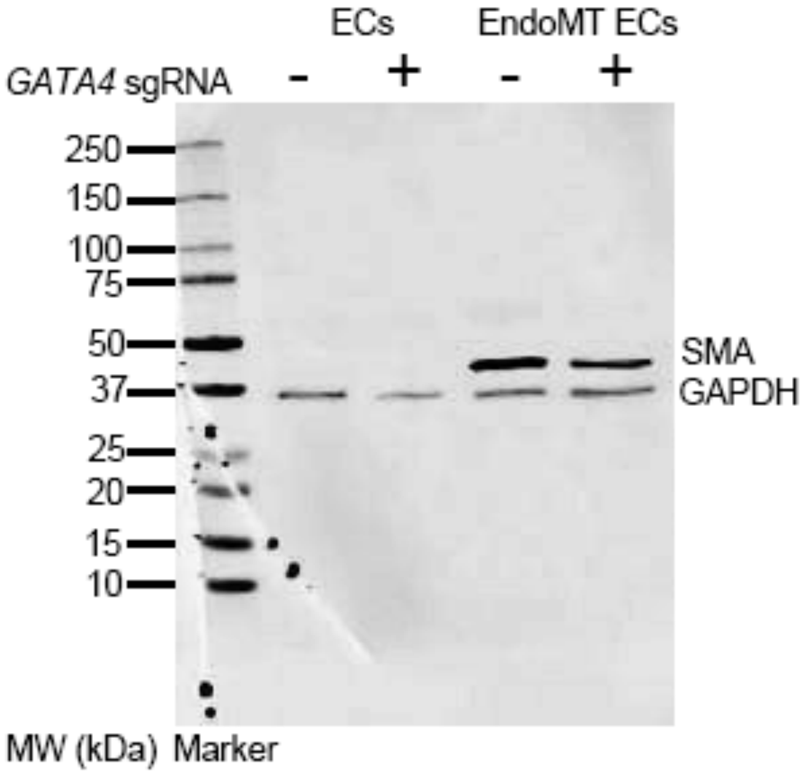

SUPPLEMENTARY FIGURE 10. Uncropped version of SMA and GAPDH western blot. This is uncropped version of Figure 3B. Abbreviations: EC: endothelial cells. EndoMT: endothelial-to-mesenchymal transition. MW: molecular weight. kDa: kilodalton.

SUPPLEMENTARY TABLE 1. Clinical characteristics of the BAV cases in the discovery cohort (n = 466)

	**BAV Cases in Discovery Cohort**
Age at inclusion, median (IQR)	39.0 (31.0-46.0)
Male sex, n (%)	345 (74)
Hypertension, n (%)	253 (54)
Dyslipidemia, n (%)	203 (44)
Smoking - ever, n (%)	198 (43)
BAV subtype, n (%)	
Type 0 anterior-posterior	6 (1.3)
Type 0 lateral	10 (2.1)
Type 1a	202 (43)
Type 1b	45 (9.7)
Type 1c	11 (2.4)
Type 2a	16 (3.4)
Type 2b	1 (0.2)
Type 2c	8 (1.7)
Type 3	5 (1.1)
No information on subtype	162 (35)
Thoracic aortic aneurysm, n (%)	
Arch	40 (8.6)
Ascending	316 (68)
Descending	10 (2.1)
Root	21 (4.5)
None	79 (17)
Aortic stenosis, n (%)	259 (56)
Aortic insufficiency, n (%)	246 (53)
Other congenital heart defects, n (%)	4 (0.9)
BAV in family*, n (%)	93 (20)

*Number (%) of cases reporting one or more family member with BAV.

SUPPLEMENTARY TABLE 2. Non-additive association results for the BAV hits in the discovery study (466 BAV cases and 4,660 controls)

Variants			Dominant Tests		Recessive Tests	
**Chr:pos**	**rsID**	**Protein Change**	**OR**	**P**	**OR**	**P**
8:11778803	rs6601627 A/G	Intergenic	2.39 (1.81-3.15)	$6.4x10^{-10}$	8.04 (1.78-36.26)	$6.7x10^{-3}$
8:11614575	exm682536 rs3729856	p.S377G GATA4	1.48 (1.21-1.82)	$1.5x10^{-4}$	1.24 (0.63-2.41)	0.5

SUPPLEMENTARY TABLE 3. Association results with thoracic aortic aneurysm (TAA) of the two BAV hits in the discovery study.

Variants			BAV with TAA				BAV without TAA				TAA without BAV				Heterogeneity P*
Chr:pos	rsID	Protein Change	Freq (%) Case/ Ctrl	N Case/ Ctrl	OR	P	Freq (%) Cases/ Ctrl	N Case/ Ctrl	OR	P	Freq (%) Cases/ Ctrl	N Case/ Ctrl	OR	P	
8:11778803	rs6601627 A/G	Intergenic	8.1/ 3.8	387/ 3870	2.29 (1.72-3.06)	$2.0x10^{-8}$	8.2/ 3.4	79/ 790	2.76 (1.41-5.40)	$3.0x10^{-3}$	4.8/ 3.6	414/ 4140	1.38 (0.98-1.95)	$6.0x10^{-2}$	0.62
8:11614575	exm682536 rs3729856 A/G	p.S377G GATA4	18.0/ 14.3	387/ 3870	1.35 (1.11-1.65)	$3.0x10^{-3}$	19.6/ 13.2	79/ 790	1.63 (1.05-2.54)	$3.0x10^{-2}$	13.7/ 13.3	414/ 4140	1.04 (0.84-1.28)	$7.2x10^{-1}$	0.44

*Heterogeneity tests were performed to compare the tests of BAV patients with TAA and the tests of BAV patients without TAA.

SUPPLEMENTARY TABLE 4. Association results with BAV cases with and without family members with BAV and/or TAA of the two BAV hits in the discovery study.

Variants			BAV cases without family members who have BAV and/or TAA				BAV cases with family members who have BAV and TAA				Heterogeneity P
Chr:pos	rsID	Protein Change	Freq (%) Cases/Ctrl	N Case/Ctrl	OR	P	Freq (%) Cases/Ctrl	N Case/Ctrl	OR	P	
8:11778803	rs6601627 A/G	Intergenic	8.5/3.6	371 / 3710	2.54 (1.90-3.41)	$4.8x10^{-10}$	6.8/4.0	95/ 950	1.84 (0.98-3.44)	$5.8x10^{-2}$	0.36
8:11614575	exm682536 rs3729856	p.S377G GATA4	18.2/14.3	371 / 3710	1.37 (1.11-1.67)	$2.7x10^{-3}$	18.4/13.6	95/ 950	1.50 (1.00-2.26)	$5.3x10^{-2}$	0.69

SUPPLEMENTARY TABLE 5. Association results with BAV subtypes of the two BAV hits in the discovery study.

Variants			BAV type 1a				BAV non-type 1a (patients without available subtype information are excluded)				Heterogeneity P
Chr:pos	rsID	Protein Change	Freq (%) Cases/Ctrl	N Case/Ctrl	OR	P	Freq (%) Cases/Ctrl	N Case/Ctrl	OR	P	
8:11778803	rs6601627 A/G	Intergenic	6.9/3.5	202/ 2020	2.07 (1.35-3.17)	$8.3x10^{-4}$	10.8/3.6	102/ 1020	3.42 (2.00-5.87)	$8.7x10^{-6}$	0.15
8:11614575	exm682536 rs3729856	p.S377G GATA4	17.3/13.4	202/ 2020	1.40 (1.06-1.84)	$1.9x10^{-2}$	15.2/14.6	102/ 1020	1.09 (0.72-1.66)	$6.8x10^{-1}$	0.34

SUPPLEMENTARY TABLE 6. Association results with BAV cases in males and females of the two BAV hits in the discovery study.

Variants			In males				In females				Heterogeneity P
Chr:pos	rsID	Protein Change	Freq (%) Cases/Ctrl	N Case/Ctrl	OR	P	Freq (%) Cases/Ctrl	N Case/Ctrl	OR	P	
8:11778803	rs6601627 A/G	Intergenic	9.2/3.8	344/3440	2.74 (2.03-3.69)	$4.0x10^{-11}$	5.3/3.5	122/1220	1.53 (0.84-2.80)	$1.6x10^{-1}$	0.09
8:11614575	exm682536 rs3729856	p.S377G GATA4	18.0/14.1	344/3440	1.36 (1.10-1.68)	$4.2x10^{-3}$	18.9/14.1	122/1220	1.48 (1.03-2.11)	$3.2x10^{-2}$	0.70

SUPPLEMENTARY TABLE 7. ICD-9 Diagnoses codes used to exclude MGI controls with aortic diseases

ICD-9	Inclusion Diagnoses for Aortic Disease MGI Controls
441	Aortic Disease - Major Classes
**Aortic dissection**	
441.00	Unspecified site
441.01	Thoracic
441.02	Abdominal
441.03	Thoracoabdominal
**Aortic Aneurysm**	
441.2	Ascending
441.1	Ascending, if ruptured
441.2	Arch
441.1	Arch, if ruptured
441.9	Descending, not otherwise specified (NOS)
441.5	Descending, if ruptured
441.2	Thoracic descending
441.1	Thoracic descending, if ruptured
441.4	Abdominal descending
441.3	Abdominal descending, if ruptured
441.7	Thoracoabdominal
441.6	Thoracoabdominal, if ruptured
441.4	Abdominal
441.3	Abdominal, if ruptured

Supplementary References

1. McCarthy S, *et al.* A reference panel of 64,976 haplotypes for genotype imputation. *Nat Genet* **48**, 1279-1283 (2016).

2. The Genotype-Tissue Expression (GTEx) project. *Nat Genet* **45**, 580-585 (2013).
